# Recent Progress on Improving the Quality of Bran-Enriched Extruded Snacks

**DOI:** 10.3390/foods10092024

**Published:** 2021-08-28

**Authors:** Catrin Tyl, Andrea Bresciani, Alessandra Marti

**Affiliations:** 1 Faculty of Chemistry, Biotechnology and Food Science, Norwegian University of Life Science, 1433 Ås, Norway; catrin.tyl@nmbu.no; 2 Department of Food, Environmental and Nutritional Sciences (DeFENS), Università degli Studi di Milano, Via Giovanni Celoria, 2, 20133 Milan, Italy; andrea.bresciani@unimi.it

**Keywords:** milling by-products, direct expanded snacks, co-extruded snacks, bran pre-treatment

## Abstract

The incorporation of milling by-products, in particular bran, into starch-based extruded snacks allows manufacturers to address two consumer demands at once, i.e., those for goods that are more sustainably produced and of higher nutritional value. However, the higher fiber content in bran than in refined cereal flours poses a limit to the amount that can be included without compromising the quality of extruded snacks, which crucially depends on expansion. Thus, several studies have focused on the effect of bran on the physicochemical characteristics of extruded snacks, leading to the need to review the recent findings in this area. Opportunities, challenges, and potential solutions of bran-enriched snacks are addressed, and several current knowledge gaps are highlighted. Specifically, the first part of the review presents the effects of extrusion cooking on bran’s compositional aspects, focusing on structural changes and product quality. After summarizing the main quality traits of extruded snacks (e.g., expansion rate, bulk density, and textural attributes), the effects of bran enrichment on the physical and sensory characteristics of the final product are discussed. Finally, bran pre-treatments as well as processing optimization are discussed as approaches to improve the quality of bran-enriched snacks.

## 1. Introduction

The global extruded snacks market was valued at USD 51.59 billion in 2019 and is expected to grow by more than 4% until 2026 as a result of changes in the eating habits of consumers and the growing demand for ready-to-eat foods [1]. Extruded snacks are convenience products and their low moisture content (around 4–6%) makes them shelf-stable. The versatility of their manufacturing process facilitates innovation in this product category, allowing the use of various ingredients and/or nutrient-rich constituents to make attractive products.

Cereal-based snack foods cover a wide range of ready-to-eat products, such as popcorn, pretzels, breakfast cereals, and expanded products. The structure and texture of such snack foods are the results of their respective manufacturing processes, which can involve operations like baking, deep-frying, or the topic of this review, extrusion cooking. This technology can be tailored to yield various products: direct-expanded (or second-generation) snacks, semi-finished product pellets (or third-generation snacks), or co-extruded products [2].

As these products are specifically formulated to allow for maximum expansion and thus starch gelatinization, extruded snacks have often been criticized for their poor nutritional profile (i.e., high starch content and glycemic index [3,4]). At the same time, extruded snack products are usually low in protein, vitamins, minerals, and dietary fiber [4]. Recently, efforts to produce extruded snacks to address these nutritional shortcomings have increased, and breakfast cereals and snacks are anticipated to be among the fastest-growing segments in the global dietary fiber market [5]. An increase in the dietary fiber content can be achieved via using: (1) Wholemeal instead of refined flours; (2) Feed mixes containing flours with high dietary fiber contents such as barley, oat, pseudocereals or pulses; (3) Bran as an ingredient; or (4) Commercial sources of purified dietary fiber preparations, typically soluble fibers such as inulin, pectin, or psyllium. The latter approach makes it possible to achieve label claims of the product being a “source of fiber” or “high in fiber” (3 versus 6 g of dietary fiber per 100 g of serving) [6] with minimal changes in formulation and product quality. The use of whole grains and/or their milling fractions (especially bran) has a positive effect on the nutrient composition by increasing the contents of protein as well as fiber and other bioactives [7]. In a recent survey of breakfast cereals available in Italy, bran-containing products were significantly higher in protein but lower in energy content than all other categories [8]. In addition, formulating foods to contain by-products addresses the crucial need for a more sustainable food production. In this context, several recent articles have highlighted compositional and functional aspects of extruded snacks formulated with by-products [9,10,11], or summarized the effects of cereal fiber on microstructural, technological, and sensory attributes of products either extruded at low (pasta) or high (snacks) temperature [12]. If a manufacturer was aiming to develop a novel bran-enriched extruded product with the help of a multidisciplinary team, a debate between nutritionists and food technologists might emerge on how much bran should be included. On the one hand, more added bran maximizes the nutritional benefits, but on the other hand, the negative impact of fiber on physicochemical features of snacks and consumer acceptability is usually evident even at low percentages (see Section 4 for a discussion on the effects of bran on extrudate quality). Almost a decade ago, Robin et al. [13] pointed out the challenges of using fiber in extrusion and discussed some approaches for suitably processing the fibers to facilitate their inclusion. Since then, several studies have explored the opportunity to include fibers either as isolated compounds or in the form of bran in extruded snacks. Researchers have expanded the range of used raw materials, such as through the inclusion of legume bran (i.e., the hulls) as well as bran pre-treatments.

**Figure 1:**
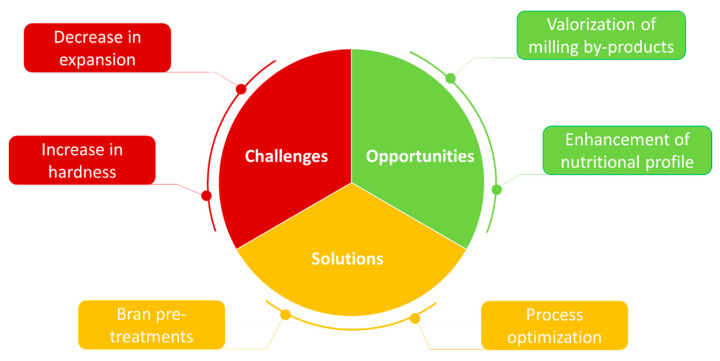
Motivations for enriching extruded snacks with bran, as well as potential issues and strategies to address them.

The focus of this review revolves around the challenges of producing bran-enriched snacks and potential solutions to assure product quality (Figure 1). This work aims to provide an overview of recent approaches for improving the features of bran-enriched snacks involving bran pre-treatment or processing optimization. It will conclude with a discussion on crucial research needs to better understand the relationship between structural attributes of the raw materials, processing conditions and product quality, so that results from different studies are easier to compare.

## 2. Effect of Extrusion on Bran Constituents

During extrusion, the feed mixture is subjected to high temperature and shear for a short time. The extrusion process is either used to modify or texturize ingredients or to produce extruded snacks. Thus, two main types of studies on bran extrusion are described in the literature, namely bran extruded to be used as an ingredient in various other products (e.g., bread) and extrudates based on whole grain flours or composite systems enriched in bran. 

### 2.1. Bran Composition

Bran is the main by-product of the dry milling of cereals. Milling separates the outer grain layers as well as the embryo from the endosperm, all of which exhibit compositional differences due to their different biological functions. For instance, proteins in the endosperm differ in both quantity, role, and functionality to bran proteins, as the former are mostly storage proteins (such as the gluten-forming proteins in wheat, which are relatively hydrophobic), whereas the latter are metabolically active and tend to be soluble in water (or saline). Other key differences are that lipid as well as enzyme contents tend to be higher in the bran than in the endosperm [14]. Moreover, bran is rich in minerals (including phosphorus, manganese, and zinc) and vitamins (E and B group) [7]. Quantitatively, the main compositional difference between the bran and endosperm is the polysaccharide profile, with the endosperms being rich in starch while bran from cereals and legumes contains large amounts of cell wall polysaccharides, most of which qualify as insoluble dietary fiber. Bran contains different cell types and thus is not homogenous in its composition. Moreover, bran from different sources contains different main dietary fiber constituents, such as arabinoxylans in wheat [14] versus mixed-linked β-glucans in oats [15]. Legume seed coats and cotyledons are high in cellulose, arabinoxylans (e.g., in lupins) or pectin (e.g., in field peas) [16]. Total dietary fiber contents in bran products span a wide range, from ca. 25% in oat bran to 88% in corn bran [13]; fiber contents may reach even higher levels in legume hulls, including up to 91.5% for the field pea [16]. Depending on the employed milling technology and the degree of refinement, a fraction of the endosperm starch may be present in the bran.

### 2.2. Impact of Dietary Fiber Types

It can be practical to distinguish dietary fiber based on solubility, i.e., differentiate between soluble and insoluble dietary fiber. However, it should be emphasized that dietary fiber encompasses a wide range of constituents, and not all soluble or insoluble fibers are alike. For instance, some types of modified starches qualify as resistant starches and insoluble dietary fiber. Such a starch would have rather different properties than, e.g., cellulose, which is also an insoluble dietary fiber. However, distinguishing between the broad categories of soluble and insoluble fibers is often done in studies on extrudates because a well-documented effect of extrusion on bran constituents is the solubilization of cell wall polysaccharides induced by hydrolysis of glycosidic bonds. This leads to a shift in the ratio of insoluble to soluble dietary fiber. However, susceptibility to hydrolysis depends on structural characteristics. For instance, sugar chains from pectin or β-glucans appear to be relatively easy to solubilize, and the latter may explain why extrudates from some barley cultivars contained more soluble fiber than the raw samples [15,17]. An increase in average molecular weight for oat and barley β-glucans upon extrusion was also reported [15,18]. Solubilization of polysaccharides increases at higher specific mechanical energy (SME) [13,19], and this parameter also affects other properties of extrudates, including the viscosity of their aqueous extracts and their water-extractable arabinoxylan contents [20]. The increased viscosity is a likely consequence of enhanced solubilization of water-unextractable arabinoxylans. Solubility of polysaccharides is influenced by their molecular weight as well as their degree of branching. A high degree of xylan backbone substitution increases solubility as it provides a steric hindrance to interactions among polysaccharide chains [21]. After extrusion, an increased ratio of xylose to arabinose was found, indicating higher contents of water-extractable arabinoxylan with a lower degree of branching [19]. In addition to increasing the soluble fraction, extrusion of lupin fiber increases the viscosity of in vitro digesta, and, accordingly, the diffusion of bile acids, which may lower cholesterol [22].

In some studies, the solubility of fibers was not significantly higher after extrusion, and differences in extrusion conditions and raw material composition among studies were proposed as the underlying reason [13]. The fact that total dietary fiber contents may decrease over extrusion has been attributed to conversion of resistant into digestible starch and generation of low molecular weight soluble fiber that cannot be analyzed with methods that solely rely on gravimetric quantification, such as official method 991.43 [23], and are lacking the chromatographic determination of oligosaccharides that is a part of newer analysis techniques, such as official method 2009.01 [24]. Some authors [25] have ascribed the observed decrease in total dietary fiber to activation of endogenous xylanase in rye, which may cleave xylose units off the arabinoxylan backbone; since monosaccharides are not quantified as dietary fiber, this would lead to lower amounts of fiber. The situation is further complicated by the fact that some researchers have found increases in total or insoluble dietary fiber, which could be due to the formation of resistant starch or (insoluble) high molecular weight conjugates derived from the Maillard reaction [26]. 

### 2.3. Impact on Minor Components

While the effect on fiber constituents is crucial for extrudate quality (see Section 4), there are numerous other effects of extrusion on bran. Processing via extrusion can positively impact the safety and quality aspects of bran, e.g., reducing mycotoxin contents [27]. In addition, extrusion treatments were shown to significantly decrease the contents of several anti-nutrients such as trypsin inhibitor, oxalic acid and phytic acid (which are accumulated in bran layers) in a range of cereal brans [28,29] and legumes [30]. The extent of the reductions varied, e.g., reductions of up to 72 and 55% of trypsin inhibitor and phytic acid could be achieved in grains, while in peas about 95% of the trypsin inhibitor activity was reduced but phytic acid only marginally decreased (by about 6%) [30]. In another study, extrudates were prepared of various rice flour to wheat bran ratios [31]. When 20% or less bran was present, complete inactivation of the trypsin inhibitor could be achieved, and thiamin and riboflavin contents increased (compared to extrudates without wheat bran). However, the content of phytic phosphorus in extrudates increased in a dose-dependent manner with bran addition, and lysine contents slightly decreased. As a consequence of antinutrient degradation, protein digestibility is improved via extrusion [31].

The effect of extrusion on micronutrients and bioactive compounds has been summarized previously [32]. While phenolic phytochemicals have been observed to degrade during extrusion [33], different classes of phenolics may differ in their susceptibility to degradation. For instance, while sorghum flavones and flavanones were almost entirely absent after extrusion cooking, some 3-deoxyanthocyanidins remained [34]. 

Phenolic compounds have received a lot of attention due to their ability to act as antioxidants. Some studies have used in vitro antioxidant assays to study the effect of bran addition to products as well as the effect of extrusion. Higher contents in some phenolic phytochemicals, notably ferulic acid (the major phenolic acid in most cereal grains) have been reported after extrusion [20,35], as a likely consequence of higher extractability due to it being released from cell walls [32]. A study on rice milling fractions observed that the phenolics esterified to cell walls decreased in bran after extrusion, with a concomitant increase in solvent-extractable phenolics [36]. The results from in vitro antioxidant assays reflected this change. However, in polished and brown rice, extrusion only led to reductions in phenolics. Moreover, quantification of individual phenolic acids via chromatography showed that the concentration in bran either decreased, increased or did not significantly change. Similar observations were made in work on wheat bran, where extrusion affected the results in some in vitro antioxidant assays, but not in others [37]. While interactions of phenolics with other constituents have been proposed to increase their stability [38], the high temperature during extrusion may lead to the degradation of some phenolics (e.g., via decarboxylation reactions [32]) and thus lower values in antioxidant assays. On the other hand, the bioaccessibility of phenolics may in fact increase due to extrusion [39,40]. Proanthocyanidins (i.e., condensed tannins) are found at high concentrations in sorghum with pigmented testa [41]. Extrusion was found to lead to a decrease in tannin contents [34,42,43] as well as their degree of polymerization [42,44]. The shift to compounds of lower molecular weight has been hypothesized to facilitate their gastrointestinal absorption [42]. Moreover, condensed tannins have been shown to form complexes with amylose, increasing the formation of resistant starch [45] and thus may lead to lower glycemic indices. In general, the glycemic index of extrudates is high due to a high proportion of starch that falls within the category of rapidly digestible [46]. Some kinds of fiber may slow down starch digestion, and this effect has mostly been reported for soluble polysaccharides that lead to high viscosity, like guar gum and β-glucans. With regard to extruded products, the presence of bran has been shown to increase slowly digestible starch at the expense of rapidly digestible starch [47]. Fiber molecules from the bran were proposed to affect digestibility due to competition with starch for water or by impairing enzymes from accessing starch [47]. However, in other studies that compared products on an equal starch basis, the hydrolysis index (representing the area under the sample’s hydrolysis curve in reference to white bread) was not affected [48] or even increased [49] when untreated rye bran was incorporated in recipes. This was contrary to the authors’ initial hypothesis and was ascribed to a less coherent structure in these products as a consequence of bran particles and more starch available for digestive enzymes. Despite of some studies finding a decrease in the hydrolysis index with increasing bran contents [50], formulating extrudates to fall within the category of a low glycemic index product would be challenging, as lower digestibility would likely only be achieved if starch gelatinization was restricted, thereby reducing product quality.

## 3. Factors Determining the Quality of Extruded Snacks and Their Evaluation

Consumer acceptance of extruded snacks crucially depends on expansion and texture, and their impact on appearance. Expansion rate is measured either by simply dividing the extrudate diameter by the die orifice diameter or assessing the product’s bulk density (i.e., the weight of a certain volume) [51]. Since the die of the extruder does not change during extrusion, the section area—measured by image analysis techniques—may be considered as an index of the degree of expansion of the product [52]. A high expansion ratio implies high porosity of the product, either due to a large number or a large diameter of gas cells. It is well-documented that expansion rate is inversely proportional to bulk density and hardness [53]. Moreover, textural properties are related to some other parameters, such as porosity, cell size and cell wall thickness, and to the final product density [13]. 

Textural properties are often evaluated by mechanical analysis, using different tests and probes such as texture profile analysis, as well as cut or shear, compression, flexural and puncture tests [54]. Regardless of the type of test, the peak force obtained (i.e., the maximum force for breaking the sample) is taken as an expression of the hardness, whereas the number of force peaks recorded during the test is often considered as an index of crispness. Texture analysis can be combined with acoustic analysis because the degree of expansion affects the frequencies of the emitted sound during eating [55]. 

Key differences between the texture of highly versus poorly expanded extrudates include that the former are perceived as crispy and light, while the latter are evaluated as harder, crunchier and less crisp by sensory panels [55]. Sensory analysis of commercial wheat- and corn flour-based extrudates indicated that product shape may elicit different scores for textural attributes; for instance, cylindrical snacks were perceived as having higher crispness and fracturability, while ring-shaped snacks were scored higher in adhesiveness and hardness [54]. In addition, studies have reported correlations between results from instrumental and sensory analysis of extruded snacks’ texture, which facilitates the use of instrumental texture analysis to predict sensory perception during product development [54]. However, sensory analysis remains a crucial tool to assess product acceptability/liking and consumer behavior. 

As highlighted by several reviews on ready-to-eat products [3,56,57,58], the physical features of snacks are strongly affected by the characteristics of the raw materials (e.g., particle size, starch damage, chemical composition, amylose content, fiber solubility, color), and processing conditions (e.g., speed, extrusion temperature, etc.). Taking into account the above, adding bran to the formulation is expected to affect the structure and thus the quality of the extruded snacks, as addressed in the following section.

## 4. Impact of Bran on the Characteristics of Extruded Snack Products

### 4.1. Impact on Expansion and Texture

**Figure 2:**
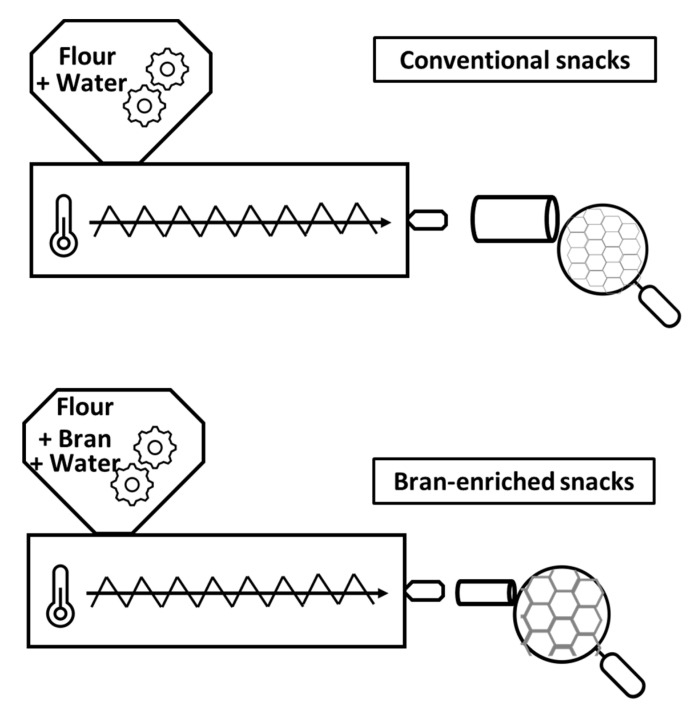
Schematic diagrams illustrating differences in shape and appearance between extruded snacks from bran-containing raw materials in comparison to conventional feed mixes based on refined flours or starches.

The main hurdle when it comes to bran incorporation into extruded products is that it restricts their expansion, making them harder, denser, and less crispy [59]. It has been suggested that bran unfavorably affects nucleation mechanisms, leading to longitudinal expansion and premature bursting of gas cells [60]. This promotes the formation of a higher number of air cells with smaller size, and reduces expansion [61], as illustrated in Figure 2.

The reduction in expansion of bran-enriched snacks has been attributed to several factors. Firstly, bran addition dilutes the starch content in the formulation, which has been recommended to be between 60–70% to ensure sufficient expansion [59]. Secondly, competition for water between fiber molecules and starch limits the extent of gelatinization [59] and water loss at the die exit, reducing the expansion of gas cells. Moreover, reduced expansion in bran-enriched samples might also be due to higher fat content favoring the amylose–lipid complex formation during extrusion, thus limiting starch swelling and gelatinization [62].

While the inclusion of bran decreased the hardness of extrudates in some studies [63], most authors reported an increase in hardness [64,65,66]. Contrasting results have been obtained for hardness of extrudates enriched with bran compared to those produced with refined flour; this is possibly influenced by processing conditions, as well as the type and amount of included bran. One study on rice bran reported that its inclusion in corn grits-based extrudates (at 10 and 15% *w*/*w*) decreased this index [63], whereas in studies using wheat (5–15% *w*/*w*) or oat (up to 50% *w*/*w*) bran, hardness increased [64,65]. Differences among the outcomes of various studies can be due to either source of bran or processing conditions. A decrease in hardness might be due to limited interactions (and/or adhesion) between polysaccharides (and proteins) when fiber is present [67,68]. On the other hand, the presence of fiber might provide greater structural rigidity, resulting in reduced sectional expansion and increased density and hardness [68].

### 4.2. Impact on Product Acceptability

The survival of a new product in a competitive market greatly depends on consumer acceptability, which in turn is influenced by consumers’ behavioral attitudes towards new food products (i.e., neophobia). Mendonca et al. [69] pointed out that panelists evaluated the acceptability of corn bran-enriched snacks (up to 32% enrichment level) much more based on texture than on appearance and that palatability was related to both fracturability and hardness [69]. However, among the physicochemical characteristics considered (i.e., specific volume, expansion rate, hardness, and fracturability), specific volume had the highest correlation with sensory attributes [69].

Proserpio et al. [70] addressed the sensory attributes determining consumer preferences regarding co-extruded snacks enriched in bran from pulses (chickpea or pea up to 30% level) [70]. A positive correlation between the hardness obtained by instrumental measurement and the “hard” attribute as evaluated by sensory analysis was shown, while negative correlations were found between instrumental hardness and crumbly, porous attributes measured by sensory analysis [70]. Moreover, the study reported that the “hard” attribute played a negative influence on consumer hedonic perception. The bran source seemed to play a role only at the higher enrichment level (30% versus 15%), with green pea and chickpea bran having the opposite impact on texture. Pea bran—being almost entirely composed of dietary fiber (92% versus 78% in chickpea bran)—could absorb water during processing, limiting its availability for starch gelatinization and thus resulting in a more compact structure with higher hardness values than the control and the extrudates with chickpea bran. At low enrichment levels (<12.5%), pea hulls had a slight deteriorating effect on the quality of the snacks, increasing vitreousness and stickiness [71].

Textural differences to their refined counterparts are not the only obstacle that needs to be overcome for bran-enriched extruded snacks to find consumer acceptance. Research on whole grain products has shown that the presence of bran affects flavor generation, and different sensory attributes are a major contributing factor to the higher preference for products made from refined grains [72]. However, compared to other cereal-based products such as bread, little is known about mechanisms of flavor generation in bran-enriched extruded snacks. Recently, it was shown that aroma differences between extruded corn puffs made from refined or whole grain flour were detected by a sensory panel as well as instrumental analysis via gas chromatography/mass spectrometry/olfactometry [73]. Intensity scores of various aroma attributes associated with the Maillard reaction were significantly higher for whole grain compared to refined grain puffs. This corresponded to whole grain puffs containing more than two times the concentration of two heterocyclic Maillard reaction products with odor descriptors that matched aroma notes for which panelists had perceived intensity differences. To the best of our knowledge, how bran addition alters pathways of flavor formation in extrudates based on other raw materials has not yet been reported. The presence of bran may also impart a bitter taste, as whole grain products are often characterized by higher bitterness than refined grain products [72]. Some strategies that have been explored for bitterness reduction, such as fermentation prior to extrusion, can also affect other extrudate characteristics. Extrusion pre-treatments are further discussed in Section 5.1.

### 4.3. Impact of Bran Composition on Attributes and Stability of Extruded Products

The negative effects on textural parameters and appearance limit the amount of bran that can be part of the feed mix without compromising on quality; in most studies, this amount was below 30% [13]. In fact, it has been proposed that when present above a ‘critical concentration’, fiber molecules prevent elastic deformation of the mix and reduce its gas holding capacity [50]. The dietary fiber content in the other ingredients will influence how much bran can be added and the addition level above which the onset of deleterious effects will occur. For instance, apparent viscosity decreased above 10% wheat bran inclusion, and this effect could not be counteracted by adjusting processing parameters [74]. Many studies have sought to determine optimum extrusion conditions (discussed in Section 5.2) for a particular feed mix composition, often through response surface methodology. However, such optimum parameters may be less effective at correcting for the effect of fiber as the bran concentration increases.

The distinction between soluble and insoluble fibers mentioned in Section 2 comes into play again when considering the effect of fiber types on quality attributes of extrudates. Contrary to enrichment with insoluble fibers, inclusion of soluble fibers did not restrict the radial expansion of extrudates in some studies [64]. The difference in expansion behavior and related textural properties between soluble and insoluble fiber can be explained by their interactions with starch and differences in water sorption and plasticization behavior, but also by the physicochemical transformations they undergo during extrusion [13]. Moreover, some soluble fibers, such as oligofructose and inulin, can be employed as extrusion aids [65], along with emulsifiers such as monoacylglycerols [69]. Pectin obtained from fruits or vegetables was observed to decrease radial expansion to a lower degree than insoluble wheat fiber [59]. However, there have also been studies where bran exhibited minor differences in total, insoluble and soluble fiber, and, e.g., radial expansion was not significantly correlated with contents of water-extractable arabinoxylans, a type of soluble fiber [75]. It was pointed out that the difference in results among some studies may be due to the wide range of evaluated soluble fiber contents (e.g., ca. 2–64% [76]}. In addition, some studies incorporated soluble fiber into the raw mix by adding hydrocolloid types that would not be present in bran (e.g., guar gum) [64] or only in low amounts compared to the contents of arabinoxylans and β-glucans in cereal bran (e.g., pectin) [77]. Nevertheless, the fact that soluble fibers tend to be less disruptive to the structure of extrudates ties into several strategies for bran pre-treatments that aim to increase soluble at the expense of insoluble fiber, discussed further in Section 5.

Compared to the endosperm fraction or wholemeal, bran is richer in lipids [78] and the activities of lipase [79], as well as lipoxygenase [14,80] are generally higher, all of which can contribute to accelerated rancidity development over processing and during storage. Several authors have therefore used defatted bran in their studies, which may also allow for more expansion (summarized by Heinio et al. [72]). A crucial parameter for how susceptible lipids are to oxidation in an extruded product is their location. Extrusion parameters are typically optimized (see Section 5.2) to allow for maximum expansion, thus creating products of large surface areas relative to their mass. A lipid molecule on the surface is directly exposed to oxygen, while location within the product matrix offers some protection [81,82]. However, surfaces of extruded products have been described to contain cracks [81,82] and their presence appears to amplify oxidation rates [81]. Aside from that, a puffed product contains air bubbles, and lipids could be present at the interface of those bubbles with the product. After cooling, an extruded product of sufficiently low moisture content enters the glassy state, where restricted molecular mobility limits oxygen diffusion [83]. However, incorporation of bran into extrudates lowers the glass transition temperature [60]; hence, extrudates may remain in the rubbery state for longer, where they are more vulnerable to oxidation. Recently, the use of saturated medium-chain triacylglycerols as coatings was shown to be a successful strategy to protect surface lipids against oxidation [84]. 

However, when carried out under appropriate conditions, extrusion may also be used to enhance the stability of bran. For instance, rice bran exhibits a particularly high activity of lipase in comparison to bran from other cereals [85]. Extrusion at a relatively low temperature can thus stabilize rice bran [86]. Among a range of treatments explored for their effect on storage stability of cereal bran, extrusion was demonstrated to lead to the lowest formation of free fatty acids in initial products as well as over storage [85]. This was attributed to the inactivation of enzymes via this treatment. Another study also observed lower free fatty acid levels over storage, with values remaining essentially constant for 60 days [87]. Extrudates based on rice starch that incorporated 10% stabilized rice bran exhibited delayed retrogradation compared to extruded rice starch controls [86]. However, the color of such extrudates was described as dark brown, which may impair incorporation into products. Extrusion of oat bran was also shown to minimize hydrolytic rancidity over storage; however, it should be carried out at a temperature sufficient to inactivate lipases (70 °C) but low enough to not induce lipid oxidation [88].

Extrusion feed mixtures typically contain the prerequisites for non-enzymatic browning reactions, most importantly reducing sugars and amines to initiate the Maillard reaction. If extrudates were produced at lower feed moisture contents, more volatile Maillard reaction products and less α-tocopherol degradation over storage in extruded rye bran were observed [89]. Prior to accelerated storage, the water activity of extrudates was adjusted to the same value. Extrudates made with fine rye bran contained significantly less α-tocopherol than extrudates containing coarse bran; however, the contents remained relatively constant over storage. These extrudates also formed more volatile Maillard reaction products over storage. The difference could be related to the fact that SME was higher in extrusion runs using finely ground bran, and that the water solubility index of these extrudates was higher, i.e., more starch hydrolysis led to more reducing ends that can participate in the Maillard reaction. Previous studies have shown that such volatile Maillard reaction products offer a certain protection against lipid oxidation [90]. However, the reduced accumulation of primary and secondary oxidation products was more pronounced when samples had been heated for extended periods of time and oxidation had progressed (e.g., to a peroxide value above 10). Moreover, the oxidation rates in the presence and absence of volatile Maillard reaction products were essentially the same if oxygen was present in excess [90]. Thus, aside from more Maillard reaction product formation, the moisture content of extrudates may also influence how fast they have reached the glassy state, as discussed above.

## 5. Strategies to Enhance the Quality of Bran-Enriched Snacks

**Figure 3:**
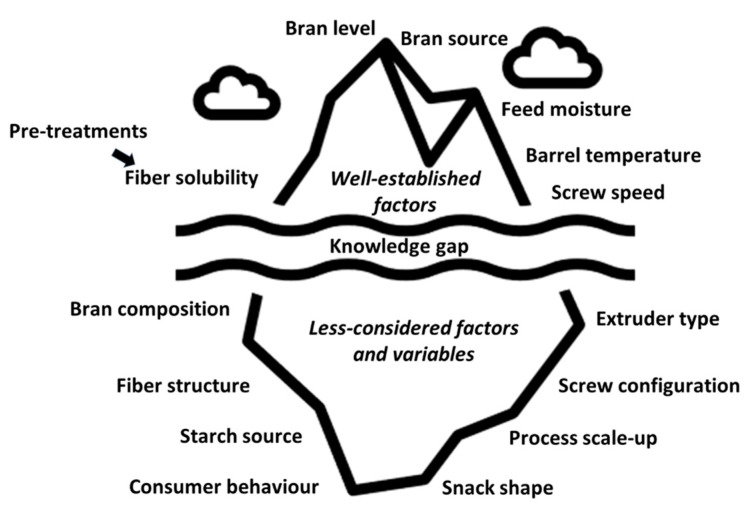
Factors and process variables most frequently investigated versus those that are rarely addressed in studies on bran-enriched extruded products. There is broad consensus among studies about the importance of factors positioned above the knowledge gap (the iceberg representing our understanding of how to manipulate the factors to counteract quality loss upon bran addition). The relationship between product quality and terms positioned below the knowledge gap remains to be elucidated by future studies.

As discussed in Section 2, bran enrichment can increase the content of protein, fiber and other bioactive compounds in snacks whose main component is starch. However, the enhanced nutritional properties come at the expense of inferior physical features of the final product. A balance between nutritional and technological quality might be achieved by adopting two approaches. On the one hand, several treatments can be used to modify the dietary fibers—mainly the insoluble fraction—prior to using the bran in food. These treatments involve mechanical, chemical and biotechnological processes. On the other hand, when a new ingredient is included in a recipe, both enrichment level and processing conditions can be optimized, and the following sections will review main findings on these approaches (Figure 3). The image symbolizes that while there is broad consensus among studies about the importance of certain factors, there is also a knowledge gap, discussed further in Section 6.

### 5.1. Bran Pre-Treatments

Pre-treatments that convert a portion of the insoluble fiber into soluble fiber may be effective in preventing the loss in product quality observed upon bran addition, as it increases physicochemical compatibility with the starch [13]. Dietary fiber can be depolymerized by the use of enzyme preparations, microbial fermentation, or addition of acids or bases. 

#### 5.1.1. Particle Size Reduction

Particle size reduction is an effective way to increase expansion and consequently decrease density. For instance, the porosity of rye extrudates was significantly higher when material of 28 μm particle size (as opposed to 143 or 440 μm) was used [91]. Finely milled rye bran especially benefited from pre-hydration, and extrudates, where this pre-hydrated bran was combined with refined rye flour/waxy corn starch (70/30) blends, were of lower density and more expanded than extrudates where water was fed to the dry mix in the barrel [50]. Pre-hydration also affected water absorption indices. However, air cell diameter and porosity were not affected, and crispiness was only improved by lower particle sizes, but not by pre-hydration. It was proposed that pre-hydration may only lead to beneficial effects on microstructure if bran is incorporated below a certain ‘critical’ percentage into the dry feed.

#### 5.1.2. Chemical Approaches

Cleavage of ester links between ferulic acid and bran polysaccharides (arabinoxylans) occurs under alkaline conditions. A combination of corn flour and corn bran that had undergone alkaline treatment gave an extruded product with significantly higher expansion than the product made from corn flour and untreated bran [76]. Combining corn flour with only the bran portion that was solubilized by the alkaline treatment increased expansion even more. A treatment like this would also disrupt cell wall architecture, which may have led to these differences, aside from the higher solubility. In addition, the use of such bran (fractions) may also lead to lower apparent viscosity during the extrusion run, allowing more bubbles to form and grow [76].

The hydrolysis of glycosidic bonds during extrusion can be promoted if acids are added to the feed mix. This has recently been shown for wheat bran extruded in the presence of 2 or 5% citric acid, where the content of water-extractable arabinoxylans increased in a dose-dependent manner (2.4 and 3.1%, respectively, versus 0.6 in the extruded bran and 1.8 in bran extruded without acid at the same moisture content) [92]. The acid-extruded samples had higher ratios of xylose to arabinose, indicating a lower degree of branching. In addition, there was a slight increase in free arabinose and a considerable increase in free ferulic acid. While the viscosity of aqueous extracts was relatively low in absolute terms, the values of acid-extruded bran were higher than for the control. Extrusion with acid led to the degradation of compounds other than arabinoxylans as well. In some instances, this led to a desirable change in terms of nutritional aspects (e.g., a decrease in phytate), while degradation of other compounds would not be beneficial (e.g., when the feed mix contained 5% citric acid, most of the fructans were hydrolyzed to monomers). Extrusion in the same conditions without acid did not lead to fructan degradation. The authors hypothesized that in comparison to regular extrusion cooking, cells would be ruptured and more disintegrated after inclusion of acid, which light microscopy images confirmed. 

A different approach to acidifying a bran sample is to incubate it with a lactic acid solution to mimic the exposure which occurs during fermentation with lactic acid bacteria [25]. This incubation procedure resulted in lower melt viscosity, product density and hardness, as well as significantly more expansion. Lower viscosity values across the whole temperature range in pasting profiles indicated starch depolymerization.

#### 5.1.3. Bio-Technological Approaches

Treatment of wheat bran with xylanase increased crispness in extrudates prepared from an 80:20 mix of refined rye flour and wheat bran [75]. Washing rice bran with water removed the majority of soluble fiber but resulted in a concentration of insoluble and total dietary fiber by removing a large portion of starch and lipids [93]. As an added benefit, phytate contents were also decreased by water washing. The washed bran was then subjected to different xylanase treatments, which resulted in the highest contents of soluble material when bran was first extruded, dried, and milled, then incubated with xylanase. However, this type of bran was not used to produce snacks.

Several studies have investigated pre-fermentation of rye bran with microorganisms that produce exopolysaccharides. Similar to added hydrocolloids like guar gum [64], these carbohydrates can improve textural properties of the so-fermented bran-containing extrudates. Additional inclusion of commercial α-amylase and xylanase further increased expansion and lowered the density, and up to 40% of refined rye flour could be substituted with bran without significantly changing these parameters compared to the control (i.e., extrudate produced exclusively from refined rye flour) [94]. The addition of these enzymes resulted in slight decreases in total dietary fiber content, which the authors attributed to hydrolysis of β-glucans and fructans, i.e., generation of low molecular weight soluble fiber that may not precipitate in 78% ethanol and thus not be determined via enzymatic-gravimetric dietary fiber measurements as in official method 991.43 [23]. In earlier work, the authors had determined side activities of the enzymes [95]. The treatments also increased cell size of extrudates.

#### 5.1.4. Other Approaches

An alternative strategy to converting insoluble into soluble carbohydrates is to use aqueous bran extracts. When prepared from defatted oat bran and used at 10–20% with oat endosperm flour, hot water-soluble material was shown to yield extrudates of superior quality compared to extrudates containing oat bran that was ultra-fine ground or treated with β-glucanase [18]. The extrudates with hot water-soluble material expanded more than all other samples, including controls prepared solely from oat endosperm flour, and had a higher crispiness index than all other bran-containing extrudates. In some studies that included pre-treatments to increase soluble fiber, non-starch polysaccharides were concentrated prior to the pre-treatment, e.g., by repeated washing with water. Such washing treatments were also reported to decrease the lipid content [93].

A reduction of lipid concentrations prior to extrusion may also be beneficial in terms of increasing oxidative stability. Defatting pre-treatments of bran remove various kinds of lipids, acylglycerols and lipophilic antioxidants, though the extent to which lipid classes are affected likely depends on the solvent used. While some studies have used supercritical fluid extraction [18], hexane is often used for commercial purposes [93,96]. The choice of solvent may affect how bran is affected by subsequent treatments. For instance, rice bran defatted with ethanol yielded higher water-soluble arabinoxylan contents after incubation with xylanase than rice bran defatted with hexane [93].

### 5.2. Optimal Extrusion-Cooking Conditions

The physicochemical and sensory characteristics of bran-enriched snacks can be impacted by controlling process conditions. The main extrusion cooking variables affecting product quality are the moisture content of the feed material, the extrusion temperature, and the screw speed. Therefore, several studies [65,69,97,98,99,100,101,102,103] have aimed to find the optimal combination of those parameters to ensure the best quality for each type of bran-enriched product (Table 1). 

Feed moisture content was highlighted as the variable with the highest influence over extrusion parameters and product properties (mainly hardness) in an optimization study [100]. Decreased moisture content increases SME and results in low density and high expansion rate [69,97,100]. The expansion rate is also affected by die temperature and screw speed: high values for both these variables produce more expanded extrudates [100]. When both temperature and screw speed were at the maximum among the evaluated conditions (i.e., 160 °C and 400 rpm), a high expansion was reached even at low moisture content [100]. 

Hardness decreases when moisture decreases or temperature increases [69]. The feed moisture-screw speed interaction was significant in a study on blue corn, yellow pea, and oat bran: at a high moisture level hardness increased with increasing speed; conversely, at a lower moisture level shear rises with increasing speed, leading to expanded extrudates with a fragile structure [100]. 

Processing optimization greatly depends on bran type, formulation, and type of extruder, as well as the range of parameters considered, making the comparison among different studies difficult (Table 1). Firstly, there is tremendous variety in the raw materials used. Some studies included an additional starch source aside from refined flour, while others included more than one by-product and/or isolated fibers. With such differences in study design, it is to be expected that few general statements regarding optimal extrusion conditions for bran-enriched snacks can be made. For example, two studies utilizing single screw extruders to process feed mixes containing rice bran (with relatively high lipid content) reached rather different conclusions on feed moisture (12 versus 30%), though both used a relatively low temperature below 100 °C [98,99]. In contrast, studies that incorporated oat bran used higher temperatures, around 160 °C [65,100] though other conditions, such as the optimum feed moisture and bran content, again differed (Table 1).

Compared to cereal bran, fewer studies have been carried out on legumes [101,102]. Based on the limited information available so far, the negative effect of legume hulls may appear at addition levels as low as 10%, e.g., leading to decreased radial expansion [102] or reduced acceptability compared to controls without hulls [103]. Extrudates containing 50–60% pea hulls were deemed too hard and compact for consumption. We are unaware of studies that have assessed quality aspects of extrudates made from pre-treated legume hulls. 

Optimization studies usually evaluate a select set of criteria, and if nutritional characteristics are considered, then the focus is often on insoluble versus soluble fiber. However, additional considerations may be valid, as several of the studies aiming to optimize extrusion parameters for bran-containing material selected a temperature on the higher end of the evaluated range and a moisture content on the lower end; both these parameters can increase SME. Such conditions may promote the formation of products with potential adverse health effects. In model systems using corn flour (not containing bran), furfural and hydroxymethylfurfural contents increased with higher temperature and lower moisture [103]. The addition of by-products, e.g., brewers’ spent grain, to corn-based extrudates increased hydroxymethylfurfural as well as acrylamide contents [104]. The contents of acrylamide in extruded products may be lower than in certain other cereal-based goods [105] but are affected by extrusion technology [105] as well as the raw material used; for example, they tend to be higher in rye than in wheat products as rye contains more free asparagine [46]. Bran pre-treatments that hydrolyze glycosidic bonds result in more reducing ends that may be available for the Maillard reaction.

## 6. Knowledge Gaps and Future Perspectives

With shifting consumer demands over the past decades, food reformulation with the purpose of enhancing products’ nutritional value has been investigated by numerous studies. In this context, bran represents a relatively widely available and economic vehicle of fiber, protein and phenolic compounds to enrich extruded snacks which are otherwise mostly comprised of starch. Although bran enrichment generally causes a decrease in expansion rate and an increase in both density and hardness, some studies showed no such effect or even the opposite trend [66,106,107]. Using different sources and percentages of bran, different grains as the main ingredient, different types of extruder and processing conditions might account for such contrasting results. Such diverse outcomes make it hard to pinpoint what results can be expected from a certain set-up, but at the same time highlight the current knowledge gaps and drivers for further research (Figure 3). The relationship between bran composition and product functionality should be better addressed and, furthermore, should be addressed in a more systematic way. The milling conditions as well as the source of bran affect its fiber, protein, starch and lipid contents, which in turn affect product features. For instance, bran from white, red or purple wheat—which are characterized by different chemical compositions—affected snack quality to different extents [66]. Taking into consideration that decorticated pulses are becoming more common in cereal-based products [108,109], the potential use of pulse bran in snack production should be further investigated.

The role that structure and functionality of bran components might play deserves to be elucidated. Information about which starch source is most suitable for bran-enriched formulations is scarce. Despite recent reports that illustrate the impact of starch characteristics, including amylose to amylopectin ratio, on the physicochemical properties of extruded snacks [52,53,110], there is a notable lack of information about why studies combined a certain bran type with a certain starch source. In other words, it is unclear if rice, corn or wheat behave in a similar way or if one of them may better counteract the negative impact of bran addition (and as to whether certain starch characteristics would influence that). Such information could take out some of the “guesswork” when using novel raw materials in future studies. However, extrusion will likely retain a certain empirical aspect in the foreseeable future.

Bran enrichment also requires a “food design” approach, which includes the assessment of those variables that mostly affect product quality and thus their optimization. Although many studies have been carried out on process optimization, their outcomes seem to be tied to the experimental plan, including the considered variables and their range of values (Table 1). In this regard, the roles of feed moisture, barrel temperature, and screw speed on snack features have been widely addressed, whereas fewer authors have pointed out the importance of screw configuration on bran functional properties [20], leaving room for further investigation on snack quality. Moreover, since most of the studies were carried out on a pilot scale, the scale-up of the process needs to be considered for large scale production of bran-enriched extruded snacks. It was recently shown that higher SME, and thus greater structural disintegration of wheat bran, can be achieved via use of an industrial scale extruder compared to pilot-scale extrusion [20]. 

Among extruded snacks, direct expanded snacks make up the majority of the products on the market, and, thus, numerous research articles have focused on this snack type. On the other hand, few studies have been published on the most recent extrusion-cooked snack category, i.e., co-extruded snacks. For such products, there are additional quality requirements, i.e., a compact outer shell and an evenly shaped hole to contain a savory or sweet filling. Regardless of the type of snack, expansion rate, bulk density and hardness are the main indices considered for quality assessment. However, other attributes that contribute to the overall quality of extruded snacks, including porosity and cell size distribution, have been poorly considered so far. Moreover, considering that textural attributes might depend on product shape [54], it should be considered if, for instance, cylindrical snacks might counteract bran enrichment better than ring-shaped snacks. Additionally, among the relatively neglected aspects of bran inclusion into extrudates are effects on flavor, which can be affected as bran (and whole grains in general) is characterized by higher bitterness [72]. Electronic noses and tongues have been proposed to successfully evaluate the contribution of different chemical species in determining aroma and tastes in wholegrain pasta and may be useful tools for extrudates as well [111]. Bran pre-treatments that use acids, enzymes or fermentation to lower the pH also affect sensory properties. Finally, in addition to evaluating the impact of bran addition on the sensory quality of snacks, studies on consumer behavior are worthy of interest. For example, the effect of fiber information on consumer’s acceptability and expectation has not been investigated for this kind of product. Establishing the right balance between the expected health benefit of eating fiber and perceived product liking might be useful to food developers to increase fiber content in snack formulations without worsening sensory attributes and pleasure [112]. In addition, food neophobia should be further investigated to define whether this behavioral attitude could impact hedonic perception. Finally, as pointed out for snacks enriched in other industry by-products [9], information on consumer behavior needs to be completed with studies focused on consumers’ willingness to pay for bran-enriched snacks. Several studies have achieved quality improvements via pre-treatments; however, these need to be balanced with their costs.

## 7. Conclusions

The nutritional benefits of including bran into formulations for extruded snack products need to be weighed against the diminished expansion and adverse effects on texture. Based on the literature on the health benefits of whole grains, it would be desirable from a nutritional perspective to incorporate bran at a level equivalent to its proportion present in the whole kernel. From a business standpoint, an amount suitable to allow for label claims of the product being a “source of fiber” or “high in fiber” may be desirable for marketing purposes.

In expanded snacks made by extrusion cooking, starch plays a pivotal role in achieving the desired product quality, as the amount and type of starch in the feed mix strongly affect the number and size of air cells that develop during extrusion. Fiber interferes with starch gelatinization and consequently affects air cell expansion, resulting in low expansion, high compactness and undesirably hard texture. In particular, the insoluble non-starch polysaccharides that are prominently represented in several bran layers restrict the amount of bran that can be included into recipes. In contrast, the presence of soluble fibers exerts less detrimental effects, which has prompted explorations on ways to best manipulate bran composition and structure to allow for enhanced expansion. However, there is a lack of systematic evaluations that provide information on how to translate the findings from one study to a different setting. As a consequence, we see the need to more thoroughly assess compositional characteristics of the bran as well as the other ingredients as an amendment to studies reporting on optimum extruder settings and feed mix composition for one particular ingredient combination. Previous work yielded valuable information by identifying variables with high impact on quality, exploring options for bran pre-treatment to enhance quality and also pointing out limits for how much bran can be added. At the same time more research is needed to address unresolved questions, such as how consumer behavior and the communication of bran’s nutritional benefits influence how bran-enriched extrudates are perceived.

## Figures and Tables

**Table 1:** The main results of the studies aimed at identifying the optimal processing conditions for the production of bran-enriched snacks. Values specified by authors as optimum (within the tested conditions) are highlighted in bold.

Source of Bran	Formulation	Bran Content (g/100 g)	Type of Extruder	Feed Moisture (%)	Screw Speed (rpm)	Maximum Temperature (°C)	Die Diameter (mm)	Quality Indices Considered for Optimization	Reference
Corn	Corn meal	18, **25**, 32	Single screw	**16**–22	-	150–**190**	-	Sensory acceptability	[69]
Rice	Rice flour (70%), corn flour (10%)	20	Twin screw	12–18 **13**	116–284 **203**	86–154 **140**	-	Lateral expansion, bulk density, water absorption index, water solubility index, hardness	[97]
Rice	Rice (86%), potato starch (2%), corn starch (8%)	4	Single screw	26.6–33.4 **30**	20.1–32.6 **26.6**	69.8–120.2 **95**	-	Texture (hardness, adhesive force, springiness, gumminess, cohesiveness)	[98]
Rice	Rice (81%), black soybean (10%)	9	Single screw	**12**–20	-	60–110 **85**	-	Expansion, color indices	[99]
Oat	Blue corn (80%), yellow pea (15%)	5	Twin screw	20–25 **18.38**	300–400 **371.98**	120–160 **158.64**	-	Porosity, hardness	[100]
Oat	Corn starch (20–30%), soy (20–50%), inulin (30–50%)	20–50 **37.5**	Single screw	23–27 **25**	-	130–**160**	-	Radial expansion, hardness	[65]
Pea	Corn grits	20–80	Twin screw	14–26 **17**	-	145–220	3.2–6	Expansion, density	[101]
Pea Oat	Corn grits, whole milk powder (0–**0.5**%)	pea: 2.5–15; ≤**7.5**	Single screw	**13.5**–16.5	-	125–175 **145**	3.5	Expansion, density, water absorption index, texture, sensory	[71]
Soybean	Corn grits	8–40 for instrumental analysis, 10–40 for sensory	Single screw	16	100–200	100–200	3	Sectional expansion, sensory, pasting parameters	[103]
